# Latitudinal gradient patterns and driving factors of woody plant sexual systems in forest communities in the Northern Hemisphere

**DOI:** 10.3389/fpls.2026.1879912

**Published:** 2026-07-03

**Authors:** Shengqian Guo, Ye Wang, Lin Wang, Di Fan, Yun Chen, Haifang Liu

**Affiliations:** 1 The Second Affiliated Hospital of Zhengzhou University, Zhengzhou, China; 2 College of Life Sciences, Henan Agricultural University, Zhengzhou, China

**Keywords:** climate change, ecological indicator, latitudinal gradient, sexual systems, woody plants

## Abstract

**Introduction:**

Sexual systems are key functional traits influencing the ecology and evolution of woody plant communities, yet their variation across climatic gradients remains insufficiently understood.

**Methods:**

We compiled data on 3,595 woody species from 30 forest dynamics plots spanning tropical, subtropical, and temperate zones across the Northern Hemisphere. Large-scale patterns were analyzed using linear regression, redundancy analysis, and structural equation modeling.

**Results:**

The proportion of monoecious species increased with latitude, whereas dioecious species decreased, revealing contrasting latitudinal trends. Climatic factors, particularly temperature- and precipitation-related variables, were the primary drivers of these patterns, with stronger climate–sexual system relationships observed in trees than in shrubs.

**Discussion:**

These results demonstrate that geographic variation in woody plant sexual systems is closely associated with climatic gradients. The consistent variation in monoecious and dioecious species across environmental gradients highlights the ecological relevance of sexual systems and suggests that they may provide useful trait-based information for understanding forest community responses to environmental change. Further long-term studies are needed to evaluate their potential monitoring value under ongoing climate change.

## 1 Introduction

The decreasing of species diversity from the equator to the Poles along a latitudinal gradient is one of the most significant biogeographic patterns on earth, which is evident in forest communities in different climatic zones (Rosenzweig, 1995). Flowers are part of a plant sexual organs and are part of the plant reproductive system. The sexual system of plants is divided into female-monoecious, dioecious, and some more complex reproductive systems (Cardoso et al., 2018; Yampolsky and Yampolsky, 1922). The sexual system influences the way plants reproduce, which in turn influences the genetic characteristics and evolutionary direction of plant populations (Barrett, 1992; Fox, 1985; Sabath et al., 2016; Wang et al., 2020a). Thus, sexual systems are important in the construction and maintenance of plant communities and ecosystems (Chen and Li, 2008; Ibarra‐Manríquez and Oyama, 1992).Sexual systems not only reflect vegetation responses to environmental change, but also influence the process of forest ecosystem evolution at a regional scale (Barrett and Harder, 1996; Charlesworth, 2006; Campbell and Kessler, 2013). Previous studies have demonstrated pronounced latitudinal gradients in plant diversity and community composition, with substantial differences among biogeographic regions such as East Asia and North America (Boufford and Spongberg, 1983; Wang et al., 2011; Chen et al., 2017). Most of these studies, however, have focused on species richness, taxonomic diversity, or functional traits, while relatively little attention has been paid to variation in plant reproductive traits at the community level. Among these traits, sexual systems play a fundamental role in plant reproduction, population dynamics, and evolutionary processes. Nevertheless, large-scale geographic patterns of woody plant sexual systems remain poorly understood, particularly across forest communities spanning tropical, subtropical, and temperate regions. This knowledge gap limits our understanding of how reproductive strategies vary along climatic gradients and respond to environmental change.

Recent evidence suggests that geographic variation in plant sexual systems is influenced by multiple biological and environmental factors, including life form, reproductive traits, climate, and plant developmental history (Chazdon et al., 2003; Queenborough et al., 2009; Wang et al., 2020b). Latitudinal gradients are widely used to examine large-scale ecological patterns because they are closely associated with systematic changes in climatic conditions such as temperature and precipitation. However, latitude itself is not a direct ecological driver; rather, it serves as a spatial proxy for multiple environmental gradients. Climatic factors may directly influence reproductive development, flowering phenology, and resource allocation, thereby affecting the occurrence and distribution of different sexual systems (Barrett and Harder, 1996; Gougherty and Gougherty, 2018; Inouye et al., 2019). In addition, environmental stresses such as drought and temperature extremes may alter sex expression and reproductive investment by influencing physiological performance and ecological interactions among plants (Etterson and Mazer, 2016; Hultine et al., 2016; Liu et al., 2021; Petry et al., 2016).For example, environmental conditions may affect the relative costs and benefits of male and female reproductive functions, alter pollination success, or influence the allocation of resources between growth and reproduction. These processes can ultimately shape the occurrence and persistence of different sexual systems within plant communities.

Consequently, geographic variation in plant sexual systems may reflect both broad-scale latitudinal patterns and the underlying environmental gradients associated with them.

Different environmental gradients reflect variation in habitat heterogeneity and resource availability. Because latitude integrates multiple climatic and ecological factors, it provides an effective framework for examining large-scale biogeographic patterns in plant functional traits. Previous studies have shown that traits associated with plant reproductive strategies exhibit systematic variation along environmental gradients (Bleher et al., 2002; Wang et al., 2020b; Xia et al., 2020). Nevertheless, whether woody plant sexual systems exhibit consistent geographic patterns across forest communities spanning tropical, subtropical, and temperate regions remains largely unknown. Addressing this question will improve our understanding of how reproductive strategies respond to environmental variation across broad spatial scales.

The relationship between sexual system and plant growth type has received the most attention (Renner, 2014; Wright et al., 2004). Previous studies have confirmed that the traits related to sexual system, such as life style, phylogeny age, and seed quality, are distributed along environmental gradients (Moles, 2018; Stephen, 1992). Therefore, the closely related plant sexual system may also show certain spatial distribution law on the environmental resource axis. A study of 491 angiosperm genera by Vamosi et al. (2003) showed that dioecious plants are more associated with woody life forms (especially trees) than sexually monoecious plants, this ecological correlation has also been confirmed in comparative studies of temperate forests in Sri Lanka and our country (Senarath, 2008; Renner, 2014; Wang et al., 2018). A study of 19,780 angiosperms in China by Wang et al. (2020a) showed that the quantitative distribution of sexual systems differed between woody and herbaceous species, and that the relative contributions of different drivers (particularly climate, evolutionary age, and mature plant height) varied among growth forms. Woody plants generally have taller canopies, longer life spans, and greater reproductive investment than herbaceous species, making them more likely to accumulate genetic changes over time. Differences in evolutionary rates may therefore contribute to variation in climate-niche evolution among growth forms (Renner and Ricklefs, 1995; Renner, 2014). Within woody plants, trees and shrubs also differ substantially in canopy height, longevity, resource acquisition strategies, and reproductive allocation. These ecological differences may lead to distinct responses of sexual-system composition to environmental gradients. Therefore, exploring the geographic distribution and environmental drivers of sexual systems in trees and shrubs will improve our understanding of large-scale biogeographic patterns in plant reproductive strategies.

Here, based on the sexual system distribution data of 3595 species in the woody plant of 30 forest communities, the large-scale trend of woody plant sexual system and its main environmental driving factors along the latitudinal gradient were explored. There are different climatic diversity and environmental gradients from low latitudes to high latitudes. The 30 forest communities we selected ranged from humid to dry climates, from plateau to plain topography, from cold temperate zones defined by seasonal temperatures in the north to tropical climates and topography in the south, this allows us to assess the geographical patterns of vegetation systems along a wide range of environmental gradients. In general, three questions were addressed: (1) What are the latitudinal patterns of plant sexual systems across the 30 forest communities?(2) How do the distributions of different plant sexual systems respond to environmental factors?(3) Do trees and shrubs exhibit similar patterns in sexual-system diversity along latitudinal gradients, and what are the main environmental factors associated with these patterns?

## 2 Materials and methods

### 2.1 Study site

**Figure 1:**
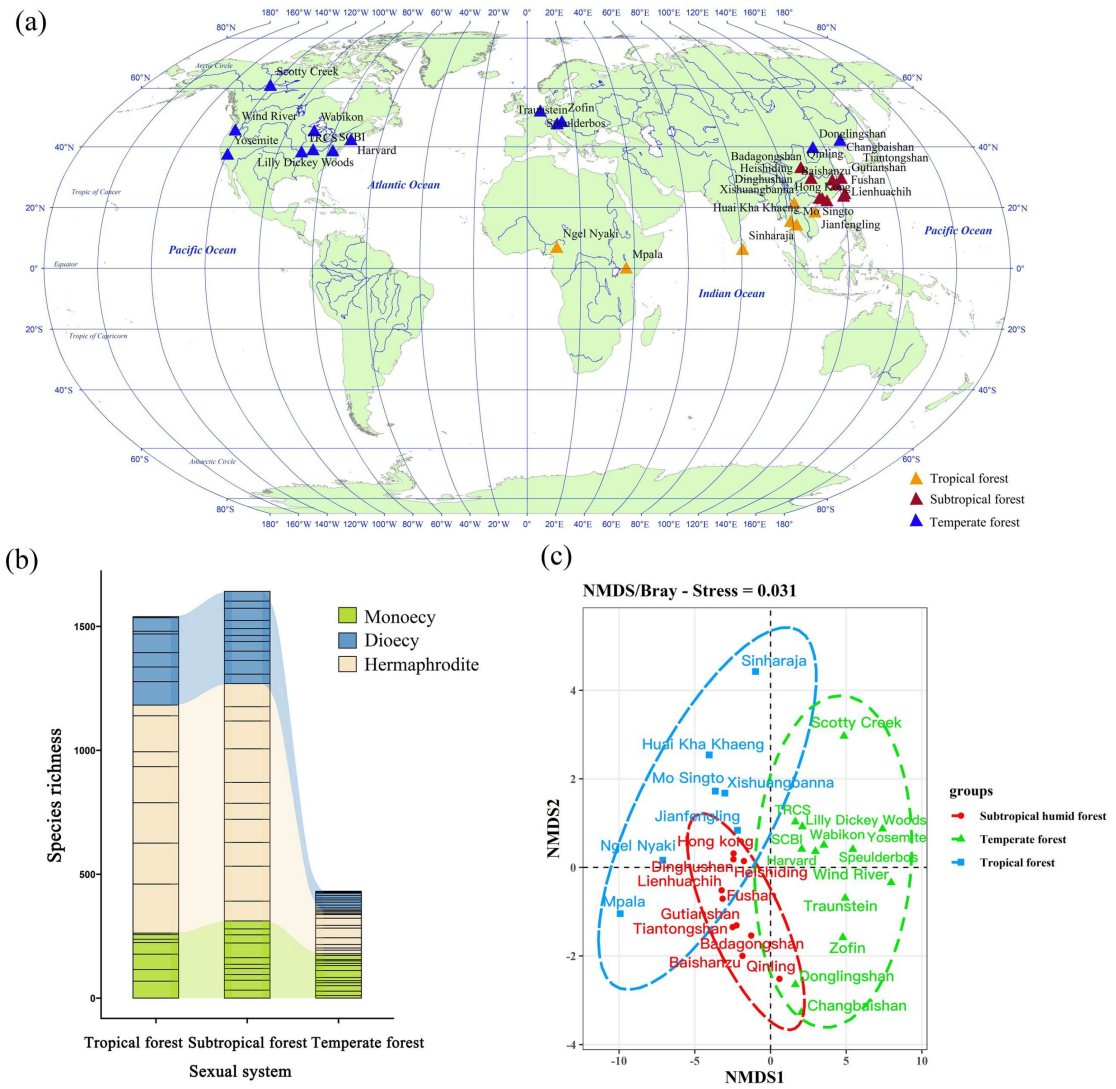
Global distribution of 30 forest plots. Plots range in size from 20 ha (Xishuangbanna) to 120 ha (Mpala) and in latitude from 0.2918°N (Mpala, Republic of the Congo) to 61.3°N (Scotty Creek, Canada), covering the continents of with Asia, Europe, and North America forests **(a)**; species richness of forest community sexual systems in different climatic zones **(b)**; NMDS analysis of species composition among thirty communities **(c)**.

Over the past three decades, the Forest Global Earth Observatory has been working on long-term, large-scale forest data for tropical, subtropical and temperate forests (ForestGEO; https://forestgeo.si.edu/what-forestgeo) (Condit, 1995). Chinese Forest Biodiversity Monitoring Network (CForBio) (http://www.cfbiodiv.org) has also expanded the CTFS network and established large mapping plots along the latitude gradient of temperate, subtropical and tropical forests.Twenty plots were obtained from ForestGEO and ten plots were obtained from CForBio. The two datasets are independent and contain no overlapping plots. All study plots were large forest dynamics plots with areas ≥20 ha.This study focused on Northern Hemisphere forest plots because comparable long-term forest dynamics plots with complete sexual-system information were limited in the Southern Hemisphere.Here, we compare the sexual-system composition of woody plants along a latitudinal gradient in 30 forest communities from these two networks (**Figure 1a**).The geographical range of the data is between 0.2918° N and 61.3000° N, with an altitude of 56 to 1902 meters. The number of species varies from 11 to 408, and the plot includes some of the most species rich forests in the world (e.g. Xishuangbanna) (Anderson-Teixeira et al., 2015; Liu et al., 2025). These macro climate and geographical differences have an impact on the change of forest plot plant sexual system. All plots we used included the number of individual trees and trunk measurements ≥ 1 cm DBH. We focused only on trees and shrubs and excluded other forms of growth (lianas, Palms).

### 2.2 Sexual systems and environment data

We obtained information on 3595 species from 30 permanent monitoring plots. In order to eliminate the species recording errors caused by differences in time, region and researchers, we checked the species names of all communities according to the *Flora Reipublicae Popularis Sinicae* (1959–2004) and *eFloras* (http://efloras.org/). Conflicting species recorded by sexual systems from different sources were repeatedly checked and corrected. The sexual systems of some species may vary depending on local biotic and abiotic conditions (e.g. climate variables or pollinator density; Barrett and Harder, 2017) (e.g. Schoen, 1982). In data analysis, we exclude them from the data set. According to the characteristics of plant life type, it can be divided into two types: tree and shrub.

The sexual system of this woody plant data set was developed by *Flora of China* (http://www.efloras.org/flora_page.aspx?flora_id=2, accessed in May 2020), *eFloras*, *Tree of Sex* (Ashman et al., 2014). According to Cardoso et al. (2018), we divided the sexual systems into three categories: hermaphroditic (species with both functional stamens and pistils in the same flower), dioecy (which refer to species with unisexual flowers in which the female and male grow on different individuals, including androdioecy, gynodioecy, and trioecy), and monoecy (which refer to species with unisexual flowers in which the male and female flowers grow on the same individual, including andromonoecy, gynomonoecy and trimonoecy).

In the study, we used data on the longitude and latitude of the plots, topographical factors [mean slope (MS, °), mean elevation (ME, m), highest elevation (HA, m), lowest elevation (LE, m)], and climate factors [mean annual precipitation (MAP, mm), Precipitation of wettest month (PWM, mm mo^-1^), precipitation of driest month (PDM, mm mo^-1^), mean annual temperature (MAT, °C), and the mean temperature of the warmest (MTWM, °C) and coldest months (MTCM, °C)]. The latitude and longitude and topographic factors are obtained from the ForestGEO (https://forestgeo.si.edu/) and the Chinese Forest Biodiversity Monitoring Network. For plots where some of these institutions do not indicate topographic factors, their topographic factors are obtained from topographic maps in the biodiversity monitoring network; and the calculation method is based on the methods of Harms et al. (2001) and Valencia et al. (2004). Climate factors are derived from the ForestGEO or literature (Anderson-Teixeira et al., 2015; Chen et al., 2016; Yuan et al., 2024).

**Figure 2:**
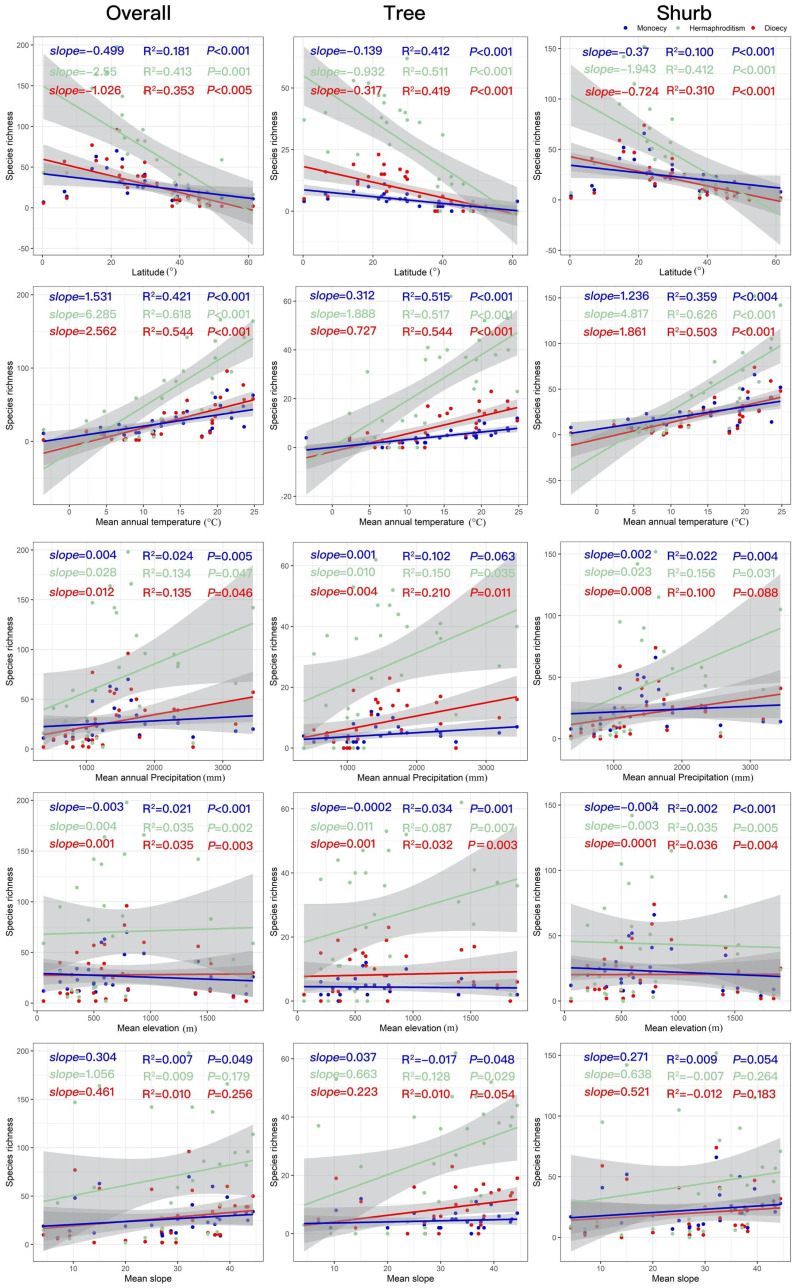
The relationship between species richness and different variables of woody plant sexual system in 30 plots. A spatial linear model with sexual system richness as the dependent variable, climate variables, topographical factors and latitude as multiple independent variables and autoregressive errors. The three rows from right to left represent all species, tree species and shrubs respectively. The five columns from top to bottom represent latitude mean annual temperature (MAT), mean annual precipitation (MAP), mean elevation (ME), and mean slope (MS).

For each forest plot, we quantified sexual systems using two complementary metrics. First, the proportional composition of monoecious, dioecious, and hermaphroditic species was calculated to characterize variation in community sexual-system structure across latitudinal gradients (**Figure 1**). Second, the species richness of each sexual-system category was used to evaluate relationships between sexual-system diversity and environmental variables, including latitude, climate, and topographic factors (**Figure 2**).

### 2.3 Data analysis

The environmental variables included in the analyses (latitude, MAT, MAP, ME, and MS) were selected because previous studies have demonstrated that climatic conditions, particularly temperature and precipitation, as well as topographic heterogeneity, are major determinants of plant reproductive traits and community composition.The proportions of species in woody plant sexual systems in the 30 forest communities were calculated based on the sexual-system data of species recorded in each forest community. The composition of woody plant sexual systems across different climatic zones was visualized using alluvial diagrams generated with the “ggalluvial” package (Brunson, 2020), and differences in species richness among sexual-system categories were presented graphically.

Non-metric Multidimensional scaling (NMDS) reflects the community differences on the multidimensional space in the form of points, and reflects the degree of differences between different places through the distance between points (Chen et al., 2018; Zhou et al., 2022). According to the stress coefficient, the goodness of fit of NMDS and the rationality of the ranking model are judged. In this study, the abundance of woody plants in 30 plots was ranked by NMDS, and the differences of species structure in forest communities in different climatic zones were displayed by fitting circles.

Linear regression analysis was used to explore the influence of latitudinal gradients and environmental factors on woody plant sexual systems. The dependent variables were the species richness of monoecious, dioecious, and hermaphroditic species, and the independent variables were environmental factors (latitude, MAT, MAP, ME, and MS). Species richness was analyzed as absolute richness and was not standardized by total plot species richness. Variance inflation factor (VIF) analysis was performed to assess multicollinearity among explanatory variables (**Supplementary Table 1**). All VIF values were below 10, indicating acceptable levels of multicollinearity. The model-adjusted R² was used to represent the explained rate of environmental variables and variables on species turnover (Tang et al., 2012). In order to avoid over-estimation caused by a large number of independent variables, we compared the values of the sample information criterion model and selected the Optimal environmental model and the spatial model respectively.

Redundancy analysis (RDA) is a ranking method combining regression analysis with principal component analysis (Borcard et al., 1992; Smith and Lundholm, 2010; Xi et al., 2021). In this analysis, the response variable matrix is the species abundance data of three woody plant systems, and the explanatory variable matrix is the environmental variable data, including longitude and latitude, topographical factors (MS, ME, HA and LE) and climate factors (MAP, PWM, PDM, MAT, MTWM and MTCM). Based on 999 permutation times, the effects of environmental factors on the distribution of community systems of woody plants were studied by using “envfit” function.

In order to further compare the influence of abiotic factors (latitude, terrain and climate) on the distribution pattern of sexual system, we generated a structural equation model (SEM) by assuming that the total effect of abiotic factors directly affects the distribution of sexual system, while latitude, MAT, PWM, MTWM and MS directly or indirectly affect the distribution of sexual system. The SEM used in this study is a statistical method based on the regression model (Grace, 2006). The complete path model includes an internal model (the relationship between potential variables) and an external model (the relationship between indicators). In this study, the potential variable is the richness of woody plant sexual system, and the observed variable is environmental factors.Covariances among monoecious, dioecious, and hermaphroditic richness were included in the SEM. These sexual-system categories jointly characterize community sexual-system structure and are not statistically independent. Bidirectional arrows represent covariance rather than causal relationships.

All analyses were conducted in R 4.0.3. (R Core Development Team^2^). Alluvial plots were generated using the “ggalluvial” package (Brunson, 2020). NMDS and RDA were performed using the “vegan” package (Oksanen et al., 2010). SEM was performed using the “lavaan” package (Rosseel Y et al., 2012).

## 3 Results

### 3.1 A pattern of sexual systems spanning latitudinal gradients

We analyzed the distribution of woody plant sexual systems across 30 forest plots spanning tropical, subtropical, and temperate regions. Across plots, the proportion of monoecious species generally increased with latitude, whereas hermaphroditic species showed the opposite pattern. Dioecious species showed relatively stable proportions along the latitudinal gradient (**Figure 1b**). These trends suggest systematic variation in sexual-system composition across different climatic zones.NMDS analysis revealed significant differences in species composition among the 30 forest communities (Bray–Curtis stress = 0.031; **Figure 1c**). Panel a of **Figure 1** illustrates the geographic distribution of the 30 forest plots, ranging from 0.29°N (Mpala, Republic of the Congo) to 61.3°N (Scotty Creek, Canada), with plot sizes ranging from 20 ha to 120 ha. Panel b presents the proportional composition of monoecious, dioecious, and hermaphroditic species across climatic zones, whereas Panel c shows differences in species composition among forest communities based on NMDS analysis.Only Northern Hemisphere plots were included in the analyses because comparable long-term forest dynamics plots with complete sexual-system information were limited in the Southern Hemisphere.

### 3.2 Effects of the abiotic factors on distributions of sexual systems richness

Species richness of hermaphroditic plants (overall: *R*^2adj^ = 0.413; tree: *R*^2adj^ = 0.511; shrub: *R*^2adj^ = 0.412) in overall plant, trees, and shrubs correlated relatively strongly with latitude and MAT compared with monoecious (overall: *R*^2adj^ = 0.181; tree: *R*^2adj^ = 0.412; shrub: *R*^2adj^ = 0.100) and dioecious plant (overall: *R*^2adj^ = 0.353; tree: *R*^2adj^ = 0.419; shrub: *R*^2adj^ = 0.310) (**Figure 2**). The significant correlation between species richness of monoecious and dioecious plants and MAT (monoecious plants: *R*^2adj^ = 0.421, *P* < 0.001; dioecious plants: *R*^2adj^ = 0.544, *P* < 0.005) was higher than that with latitude (monoecious plants: *R*^2adj^ = 0.181, *P* < 0.001; dioecious plants: *R*^2adj^ = 0.343, *P* < 0.001). The species richness of the three sexual systems was significantly negatively correlated with latitude, while the species richness was significantly positively correlated with the MAT (**Figure 2**). From the perspective of life forms, the sexual system species richness of trees and shrubs has the strongest correlation with latitude and MAT, followed by MAP, and the weakest correlation with slope and ME.Relationships between species richness and topographic variables (mean elevation and mean slope) were generally weak compared with climatic variables, although some significant associations were detected in particular sexual systems and growth forms (**Figures 2d, e**).

**Figure 3:**
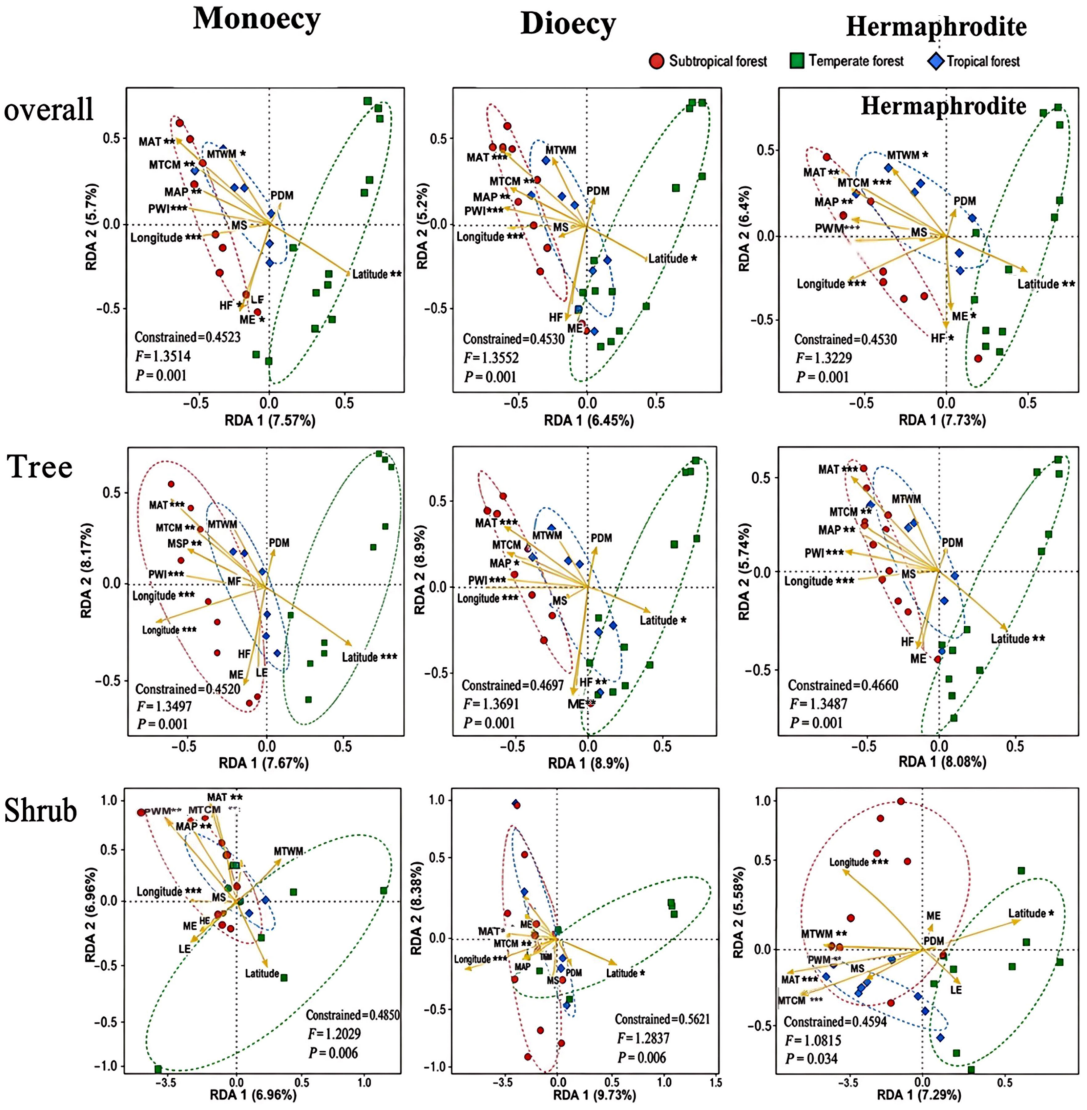
Ordination plot of monoecious, dioecious and hermaphroditic communities from redundancy analysis (RDA) among different plant life forms. (The ellipses are the 95% confidence interval. “*” represents The relationships between the community and environmental factors. “* * *” indicates the strongest correlation (*P* < 0.001), “**” indicates the strong correlation (*P* < 0.01), “*” indicates the correlation (*P* < 0.05). MS, mean slope; ME, mean elevation; HA, highest elevation; LE, lowest elevation; MAP, mean annual precipitation; PWM, precipitation of wettest month; PDM, precipitation of driest month; MAT, mean annual temperature; MTWM, mean temperature of the warmest month; and MTCM, mean temperature of the coldest month.).

For monoecious, dioecious and hermaphroditic plants, environmental variables explained 7.57%, 8.45% and 7.37% of the changes in sexual system combination structure, respectively. RDA ordinating showed that longitude and MAT were most important factors for monoecious, dioecious and hermaphroditic plants. At the same time, PWM is also an important factor affecting the three plant sexual systems. The more important factors affecting the tree and shrub plant sexual systems are climate factors and longitude, respectively (**Figure 3**).

### 3.3 The effects of climate on the sexual systems richness

**Figure 4:**
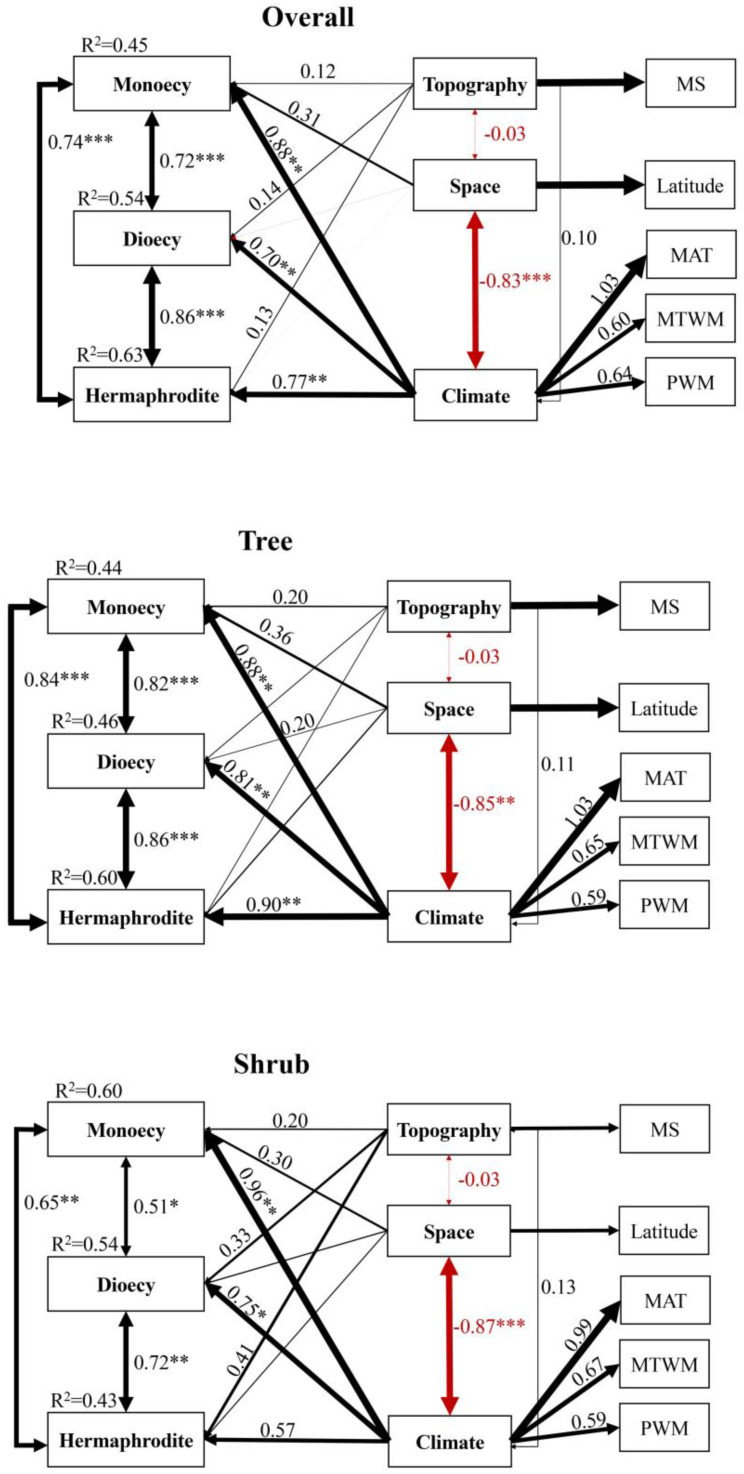
The path map shows the interaction between climate [temperature (MAT), precipitation (MTWM and PWM)], topography (MS), space (latitude) and sexual system richness. The value next to the arrow is the normalized path coefficient. Red values indicate negative correlation and blue values indicate positive correlation. The width of the path arrow line indicates the strength of the relationship. “* * *” represents the path with *P* < 0.001, “* *” represents the path with *P* < 0.01 and “*” represents the path with *P* < 0.05.

In the SEM model, the climate factors (MAT, MTWM, PWM) are positively correlated with the species richness of the three sexual systems, and the path coefficients of monoecious (0.88), dioecious plant (0.70) and hermaphroditic plant (0.77) are greater than those of the three sexual systems with topographical and space factors (**Figure 4**). For different life forms of plants, the path coefficient of climate on arboreal dioecious (0.88) is lower than that of shrub (0.96), while the path coefficient of shrub dioecious (0.81) and hermaphroditic flower (0.90) is higher than that of arbor (0.75; 0.57) (**Figure 4**). SEM results show that the sexual system modules respond differently to environmental variables. Compared with the dioecious module, the relationship between the monoecious module and environmental variables is more significant.

## 4 Discussion

Our study revealed a clear latitudinal trend in species richness of different plant sexual systems in different regional forest communities. These large-scale patterns varied among sexual-system categories and between trees and shrubs. Climatic variables explained a substantial proportion of this variation.

### 4.1 The diversity of plant sexual system changes with latitude gradient

In this study, we compared the proportions of different plant sexual systems in forests along latitudinal gradients in the tropics, subtropics, and temperate zones. The results showed that the proportion of hermaphrodite species along the latitudinal gradient did not increase or decrease significantly in East Asia and North America. The proportion of dioecious species increased with the decrease of latitude. The trend contrasts between monoecious and dioecious species proportions of these sexual systems produce strong geographic differences along the overall latitudinal gradient (Ramírez, 2005; Ramirez and Brito, 1990; MaChado et al., 2006). Overall, these results indicate that the monoecious species proportion is proportional to the latitude gradient, while the dioecious species proportion is inversely proportional to the latitude gradient. To some extent, it is revealed that the proportion of monoecious and dioecious species may be closely related to the region, especially to the latitude gradient. However, the proportion of hermaphroditic plants did not show obvious regularity along the latitudinal gradient (Chen and Li, 2008).

Early studies found higher numbers of dioecious species than monoecious species in tropical regions at low latitudes (Freeman et al., 1979; Sakai et al., 1995; Matallana et al., 2005). Nathan and Muller (2000) also confirmed our findings that many tropical low-density tree species have a higher proportion of dioecious species. But recently Wang et al. (2021) used nearly 70,000 angiosperm species to explore the spatial distribution patterns of angiosperm sexual systems on a global scale, it is confirmed that both dioecious and monoecious species have higher frequency at high latitudes. Other studies have found that dioecious species are more common at high latitudes in the Northern Hemisphere, and studies in North and Siberia have found that, the proportion of dioecious species increases with latitude (Fox, 1985; Freeman et al., 1979; Godin, 2014; Kevan and Godglick, 2017). The current spatial pattern of sexual systems in angiosperms is due to wider environmental tolerance and stronger dispersal ability of dioecious and monoecious plants. The findings may also be relevant to the fact that the data we used were the first species census data for these large plots, and therefore are consistent with earlier studies by others (Chen and Li, 2008; Nathan and Muller, 2000; Gross, 2005).

### 4.2 Effect of climate on sexual systems richness

In the field of plant reproductive ecology, plant sexual systems and the mating patterns they determine are highly sensitive to changes in climate and geographic environments (Hedhly et al., 2009; Eckert et al., 2010; Shimizu et al., 2011). Our results show that the correlation between hermaphroditic plants and latitudes and MAT is stronger than that between monoecious plants and dioecious plants, and the tree and shrub of the three sexual systems have the strongest correlation with latitudes and MAT (**Figure 2**). Climatic conditions (temperature and precipitation) can also influence the expression of sexual system status by altering the physiological and ecological characteristics of plants, by regulating their reproductive performance during flowering (Charlesworth, 2006; Weeks et al., 2014). Our results suggest that variables representing environmental temperature and precipitation were selected in the best models explaining the proportion of three sexual systems species for all species, for tree species and for shrub species (**Figure 4**). In addition, although topographic environment, MAP, PDM, and MTCM variables may also affect geographic variation in the proportion of sexual systems of all species, trees, and shrubs, respectively, their effects are weaker than those of MAT and PWM. These findings suggest that climate factors should be the main drivers of the composition and diversity of sexual systems. This may be due to ongoing climate changes (such as decreased precipitation and higher winter temperatures) and changes in plant phenology (such as early flowering) (Etterson and Mazer, 2016; Liu et al., 2021), the differences in gender resource demand further affect the population structure and the local composition and even geographical distribution of the diversity of sexual systems (Kay and Picklum, 2013; Etterson and Mazer, 2016; Wang et al., 2020b).

Other findings confirm studies on the determinants of species composition in forest community systems at different scales (Ramírez, 2005; Wang et al., 2020b, 2021). For example, a recent study on the effects of environmental stress on the sex ratio in angiosperms showed a significant correlation between monoecious and dioecious species ratios and climatic factors (temperature and precipitation) (Varga and Soulsbury, 2020). In summary, our results confirm recent findings that temperature and precipitation are major drivers of sexual-system distributions in woody plant communities. They also suggest that spatial effects should not be ignored when interpreting the factors shaping sexual-system composition.

Our results therefore suggest that sexual systems are not merely evolutionary traits but also functional ecological indicators that integrate plant reproductive strategies with environmental conditions. Given their sensitivity to temperature and precipitation, monitoring the shifts in monoecious and dioecious species frequencies could provide valuable insights into how forest communities are responding to ongoing climate change. This highlights the potential of plant sexual systems as a novel, trait-based metric for biodiversity monitoring under global warming.

In addition, species richness of sexual-system categories was analyzed as absolute richness rather than being standardized by total plot species richness. Consequently, some observed geographic patterns may partly reflect variation in overall community diversity among plots in addition to changes in sexual-system composition. Future studies incorporating richness-standardized metrics or relative abundance measures may help disentangle these effects.

### 4.3 Effect of the abiotic factors on growth form of sexual systems richness

The results of SEM model show that the significant correlation between climatic and topographical factors and the three sexual system of trees is stronger than that of shrubs (**Figure 4**). Consistent with previous large-scale studies (Oberle et al., 2009; Wang et al., 2009). Woody plants generally have longer life spans and larger body sizes. Their aboveground tissues remain exposed to climatic conditions for extended periods, potentially increasing their sensitivity to long-term environmental variation (Wang et al., 2020a). Therefore, climate has a significant impact on the spatial pattern of woody plants compared to shrubs (Currie and Paquin, 1987). These results suggest that differences in the effects of climate on the geographic patterns of the sexual systems of woody and shrubs species may also be related to differences in ecophysiology strategies for resource acquisition and utilization between different growth forms.

One limitation of this study is that phylogenetic relatedness among species was not explicitly incorporated into the analyses. Plant sexual systems are often phylogenetically conserved, and closely related taxa may share similar reproductive strategies due to common evolutionary history. Therefore, some of the observed geographic patterns may partly reflect differences in lineage composition or historical biogeographic processes among forest communities rather than environmental filtering alone. Future studies integrating phylogenetic information would help disentangle the relative contributions of evolutionary history and environmental factors to large-scale variation in woody plant sexual systems.

### 4.4 Potential monitoring value of plant sexual systems

Our findings suggest that sexual-system composition, particularly variation in the proportions of dioecious and monoecious species, may serve as a useful trait-based indicator of environmental conditions. Monitoring changes in sexual-system composition may provide complementary information for long-term forest monitoring programs such as ForestGEO and CForBio.Because the distribution of different sexual systems was significantly associated with climatic variables, monitoring changes in sexual-system composition may provide complementary information on how forest communities respond to environmental variation. However, the present study is based on spatial associations across climatic gradients rather than temporal observations, and therefore does not directly evaluate the performance of sexual systems as indicators of ongoing climate change. Compared with traditional approaches such as remote sensing, which often focus on canopy greenness or biomass, sexual-system composition may provide additional insights into plant reproductive strategies and community assembly processes. Nevertheless, large-scale monitoring of sexual systems requires long-term field observations and taxonomic expertise, which may limit its application in rapid assessments. Future studies integrating sexual-system monitoring with remote sensing and trait-based databases may provide a more comprehensive framework for tracking biodiversity responses to environmental change.

## 5 Conclusion

The geographic distribution of the frequency of the woody plant sexual system along the latitudes of 30 communities in different regions was mapped, and the effects of the abiotic component on the frequency of the woody plant sexual system were compared. The results showed that the frequency of monoecious and dioecious plants changed with the change of latitudinal gradient. There are significant differences between woody and shrub plants in the frequency and determinants of their sexual systems. These findings reveal about the relationship between forest community sexual systems and environmental factors along latitudinal gradients. Taken together, our study not only uncovers large-scale geographic patterns of woody plant sexual systems but also underscores their application as practical ecological indicators for assessing and predicting climate-driven ecosystem changes. Incorporating sexual system dynamics into long-term forest monitoring frameworks may significantly enhance our ability to detect, interpret, and mitigate the impacts of climate change on biodiversity.

## Data Availability

Publicly available datasets were analyzed in this study. This data can be found here: https://forestgeo.si.edu/what-forestgeo.

## Supplementary material

Supplementary Table 1 Variance inflation factor (VIF) values of explanatory variables used in the regression models.
