# Supplementary material for: Latitudinal gradient patterns and driving factors of woody plant sexual systems in forest communities in the Northern Hemisphere
Source: Front Plant Sci. 2026 Jul 3;17:1879912. doi: 10.3389/fpls.2026.1879912 (PMC13376281; doi:10.3389/fpls.2026.1879912)
Supplement: Supplementary Table 1 — Variance inflation factor (VIF) values of explanatory variables used in the regression models. [file Table1.pdf]

Table S1. Variance inflation factor (VIF) values of explanatory variables used in the regression models.

Variable	VIF
Latitude	3.92
MAT	7.55
MAP	8.69
ME	2.76
MS	8.96
